# Acquired Hemophilia A Masquerading as Bleeding on Anticoagulation: A Case Report Including Key Laboratory Considerations

**DOI:** 10.7759/cureus.41029

**Published:** 2023-06-27

**Authors:** Michael MacNeill, Eman M Mansory, Alejandro Lazo-Langner, Chai W Phua

**Affiliations:** 1 Medicine, Schulich School of Medicine & Dentistry, Western University, London, CAN; 2 Hematology, King Abdulaziz University, Jeddah, SAU; 3 Hematology, London Health Sciences Centre, London, CAN; 4 Hematology, Schulich School of Medicine & Dentistry, Western University, London, CAN

**Keywords:** partial thromboplastin time, hemophilia a, hematoma, blood coagulation tests, anticoagulants

## Abstract

We report a case of a patient with recurrent hematomas while on anticoagulation for a pulmonary embolism and a prolonged hospital stay due to a delayed diagnosis for acquired hemophilia A. Acquired hemophilia A is a rare autoimmune bleeding disorder with autoantibodies directed against coagulation factor VIII (FVIII), leading to an acquired FVIII deficiency. A prolonged isolated activated partial thromboplastin time (aPTT) in a bleeding patient warrants workup for acquired hemophilia A. This is specifically challenging in patients with thrombosis on anticoagulation and can lead to significant delays in diagnosis and associated morbidities. The case highlights the need for further awareness of this disease, potential laboratory pitfalls when conducting and interpreting coagulation assays, and the management considerations in a patient with a simultaneous thrombotic and hemorrhagic condition.

## Introduction

Acquired hemophilia A is an autoimmune bleeding disorder caused by autoantibodies directed against coagulation factor VIII, leading to an acquired FVIII deficiency [1]. Acquired hemophilia A is a rare condition, with the incidence estimated to be 1.5 cases per million per year [2]. About 50% of acquired hemophilia A cases are idiopathic; the rest can be associated with other etiologies, including the peri/postpartum state, autoimmune disorders, medications, infections, dermatologic conditions, and malignancies [3].

Patients with acquired hemophilia A often present with extensive ecchymoses, which can be spontaneous or secondary to minimal trauma [3]. Compared to those with hereditary hemophilia A, acquired hemophilia A patients present later in life, often over 50 years, rarely experience hemarthroses and have no male predominance nor significant personal or family history of bleeding diathesis [2]. An isolated prolonged activated partial thromboplastin time (aPTT) that does not correct with a mixing study after incubation at 37°C for two hours is often the diagnostic clue for acquired hemophilia A, as anti-FVIII antibodies are both time and temperature dependent [4]. The confirmatory test includes a low FVIII activity with an inhibitor to FVIII measured through the Bethesda assay. Management of acquired hemophilia A consists of a three-prong approach with bleeding control, often with a bypassing agent, elimination of the inhibitors via immunosuppression, and management of the underlying cause if identified [2]. We present a case demonstrating the need for a high clinical suspicion for acquired hemophilia A in a patient with recurrent bleeding while on anticoagulation for a pulmonary embolus, including the unique challenges from a laboratory perspective.

## Case presentation

**Figure 1:**
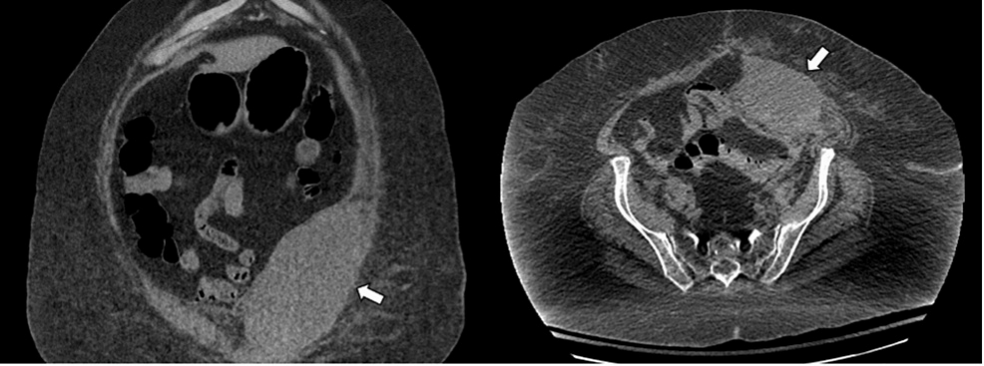
Large Rectus Sheath Hematoma Coronal and axial CT of the abdomen shows a large left rectus sheath hematoma measuring 17.2 x 9.3 cm which deviates the bladder toward the right (arrow).

A 70-year-old Caucasian female with a complex medical history including heart failure, chronic obstructive pulmonary disease, and a body mass index (BMI) of 58 kg/m^2^ presented to the hospital after a fall. On history, she reported two months of increasing shortness of breath and no previous personal or family history of bleeding. Initial investigations demonstrated normocytic anemia of 65 g/L requiring transfusion. The patient’s hemolytic workup was negative, and she completed an upper and lower endoscopy which did not identify any active source of gastrointestinal bleeding or malignancy. Despite transfusion, the patient remained dyspneic and a ventilation-perfusion scan was ordered instead of a CT pulmonary angiography due to an acute kidney injury which revealed findings compatible with a pulmonary embolism. As a result, her prophylactic dalteparin dose was increased to a therapeutic dose of 30,000 U subcutaneously daily (200 U/kg dose). Two days later, the patient developed worsening abdominal pain and a drop in her hemoglobin from 72 g/L to 58 g/L. CT abdomen revealed a large rectus sheath hematoma, left hemipelvis extraperitoneal hemorrhage, and a small right psoas muscle hematoma (Figure 1). Her bleeding was attributed to dalteparin and immediately suspended, and an inferior vena cava filter was placed. Her aPTT was prolonged at 36 secs (normal range: 20-29s), but no further investigations were pursued. Once her hemoglobin stabilized with no further evidence of bleeding, her dalteparin was reinitiated at 5,000 U and titrated up to the target therapeutic dose over 14 days to a therapeutic dose. She was then discharged to a rehabilitation hospital.

A month later, she developed an acute drop in her hemoglobin secondary to a large left calf hematoma (12.8 x 4.6 x 5.2 cm) which necessitated transfer to an acute hospital and further transfusions. This bleeding was again attributed to her anticoagulation. As such, her dalteparin was held until there was no further active bleeding and then reinitiated and slowly increased to therapeutic dosing before being discharged home after a prolonged hospital stay. No aPTT was sent on that admission.

Unfortunately, she required a second re-admission a month later when she presented with a hemoglobin of 74 g/L and was found to have a large gluteal hematoma. Her anticoagulation was again held. Coagulation assays were sent, and her aPTT was prolonged at 44 secs with a normal international normalized ratio (INR), but no action was taken until a repeat test a week later revealed further prolongation of her aPTT to 67 secs. Hematology was then consulted, and further workup revealed an FVIII of 0.04 U/mL (reference range: 0.50 - 2.00 U/mL) via a one-stage factor activity assay and an FVIII inhibitor titer of 5.6 Bethesda Units (reference range: <0.6 BU). Von Willebrand testing revealed an antigen level of 4.12 IU/mL (reference range: 0.50 - 2.00 U/mL) and activity of 2.56 U/mL (reference range: 0.48 - 1.73 U/mL).

Additionally, the presence of low-molecular-weight heparin (LMWH) was ruled out with an anti-Xa level of <0.10 U/mL. Her lupus anticoagulant (LA) testing was challenging to interpret (IgG and IgM antibodies to anti-cardiolipin and anti-β2-glycoprotein-1 were negative). As such, we completed a chromogenic FVIII activity assay to rule out the potential for confounders, such as a non-specific inhibitor from LA, especially with her concurrent acute thrombosis. The results were concordant with the one-stage FVIII activity measuring 0.04 U/mL. Further investigations with serum protein electrophoresis revealed an IgM kappa monoclonal protein of 3.2g/L. These results were consistent with acquired hemophilia A, likely from an underlying lymphoproliferative disorder due to the findings of an IgM kappa paraproteinemia. CT body was not remarkable for underlying lymphadenopathy nor malignancy.

**Figure 2:**
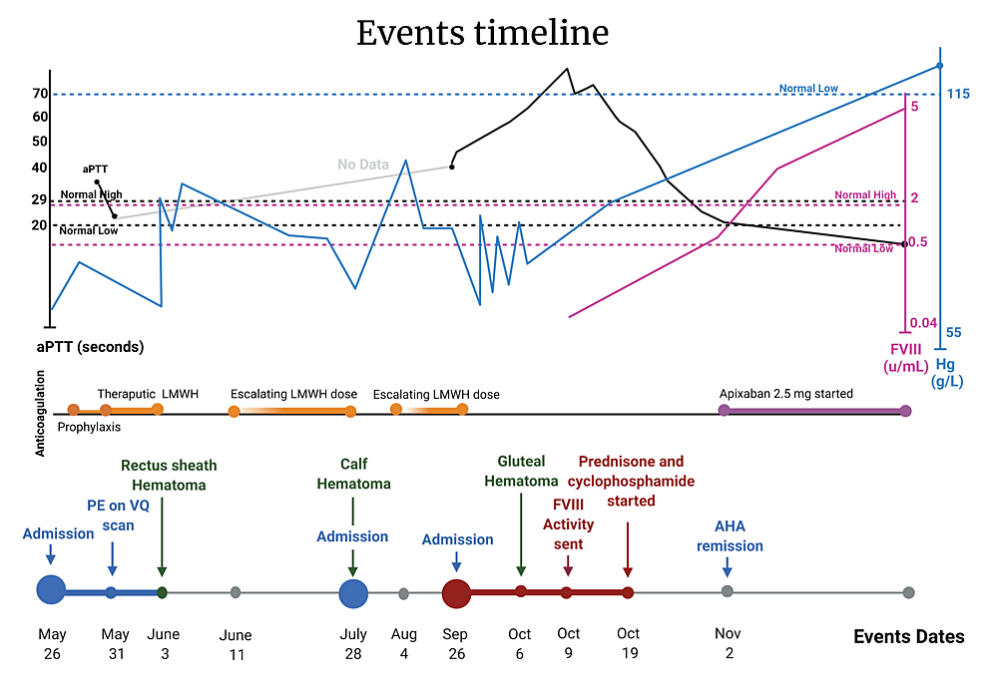
Laboratory Investigations According to Timeline of Events Shown are the hemoglobin levels (Hb; in blue), activated partial thromboplastin time (aPTT; in black), factor VIII activity (FVIII; in red) with the timeline of clinical events in our patient with acquired hemophilia A masquerading as an anticoagulation-related bleed. AHA: acquired hemophilia A, aPTT: activated partial thromboplastin time, FVIII: factor VIII, PE: pulmonary embolus, VQ: ventilation-perfusion.

The patient was started on immunosuppressive therapy with prednisone 100 mg (1 mg/kg) and cyclophosphamide 100 mg (1 mg/kg) orally once daily. Due to concerns for additional thrombotic events, bypassing agents were not empirically initiated, and she was monitored closely for bleeding events during her length of stay. After about four weeks, her FVIII activity normalized to 0.72 U/mL without a quantifiable FVIII inhibitor, consistent with at least a partial remission (Figure 2), which led to the discontinuation of her cyclophosphamide and tapering of her prednisone over about nine weeks. Her anticoagulation was restarted at the lower dose of apixaban 2.5 mg orally twice daily for her unprovoked pulmonary embolism.

The patient is undergoing active follow-up within the hematology clinic for monitoring for relapse of her acquired hemophilia, management of her pulmonary embolism, and active surveillance of her IgM paraproteinemia. She has remained relapse-free for about 20 months since her diagnosis without evidence of recurrent thrombosis or new bleeding complications.

## Discussion

This complex case underscores the importance of thoroughly evaluating for other causes of bleeding in a patient with recurrent bleeding while on LMWH. In retrospect, the initial prolonged aPTT warranted further evaluation which could have prevented her multiple admissions, transfusion of 15 units of packed red blood cells, and the diagnostic delay of about four months. Advanced age is an important risk factor for anticoagulant-associated bleeding [5]; however, the overall rate of LMWH-related major bleeding is rare and estimated at 2.5 of every 1,000 patients [6]. The patient was started on a therapeutic LMWH with dalteparin at 200 U/kg, which exceeded the recommended maximum dose of 18,000 U/day, but therapeutic LMWH dosing based on body weight is recommended and has not been shown to increase the risk of bleeding significantly [7]. Additionally, while not performed in this case until the patient was off LMWH, anti-factor Xa monitoring has been suggested to assess the clinical effectiveness of LMWH, particularly in patients with obesity and unexpected bleeding or concerns of impaired renal clearance [7]. So, while measuring anti-factor Xa is generally not recommended, and there is a lack of clear evidence for its efficacy, it could be considered for LMWH monitoring in this case [7]. Furthermore, the pattern of bleeding with varying sites of involvement and predominance of spontaneous soft tissue/muscular hematomas was peculiar and reminiscent of acquired hemophilia A, which primarily presents in such a manner [2]. A search for an acquired bleeding disorder requires a systematic approach with first steps including a detailed medical history emphasizing bleeding and family history, a physical examination to rule out secondary conditions, and initial laboratory-screening tests such as a complete blood count, INR, aPTT, fibrinogen, and a blood smear [8]. We suspect that the initial prolonged aPTT in a bleeding patient could have been a diagnostic clue as LMWH therapy does not substantially prolong the aPTT [9]. When assessing a patient’s anticoagulation assays, it is vital to understand the expected effect that anticoagulants can have on these assays to allow for accurate interpretation (Table 1) [10].

**Table 1:** Expected Anticoagulation Effects on Coagulation Assays Common classes of anticoagulants, including vitamin K antagonists (VKAs), low-molecular-weight heparin (LMWH), unfractionated heparin (UFH), anti-Xa inhibitors and anti-II inhibitors, and their effects on various coagulation assays, including activated partial thromboplastin time (aPTT), international normalized ratio (INR), Clauss Fibrinogen and anti-Xa activity. Where two arrows (↑↑) represent a significant increase above normal, one arrow (↑) represents a mild increase, and a dash (–) represents no effect.

Drug Class	aPTT	INR	Fibrinogen (Clauss)	Anti-Xa Activity
VKA	↑/–	↑↑	–	–
LMWH	↑/–	–	–	↑↑
UFH	↑↑	–	–	↑↑
Anti-Xa inhibitors	↑/–	↑/–	–	↑↑
Anti-II inhibitors	↑↑	↑/–	–	–

This case presents unique challenges with coagulation assays, as it is imperative to recognize possible confounding factors for coagulation assays in determining a true FVIII deficiency. Firstly, the presence of an LA warrants specific consideration, especially in a patient presenting with a pulmonary embolus and prolongation of the aPTT. LA can interfere with phospholipid-dependent coagulation tests such as the LA-sensitive aPTT (prolonged), the one-stage factor activity assays (falsely low), and the Bethesda inhibitor assay (false positive inhibitor) [11]. Hence, understanding your local laboratory aPTT assays and determining whether an LA-sensitive or LA-insensitive reagent is used could help narrow your differential diagnosis. We suspect that the presence of LMWH and the autoantibody to FVIII interfered with our LA testing making it challenging to interpret in this case. Therefore, we performed a chromogenic FVIII activity assay that utilizes high-test plasma dilutions, circumventing any effects from LAs, other antiphospholipid antibodies, heparin, low-molecular-weight heparin, or lupus inhibitors which may interfere in the one-stage aPTT-based factor activity assay [12]. Other considerations including enzyme-linked immunosorbent assays for detecting anti-FVIII antibodies should be considered if an interfering LA is present [13]. Utilizing a 50/50 mixing study of patient and normal plasma can also differentiate the presence of LA or a non-specific inhibitor as the anti-FVIII antibody is both time and temperature dependent [4].

Additionally, it is also prudent to recognize the effects of the patient’s apixaban (anti-Xa inhibitor) on common coagulation assays as it could lead to spurious results affecting coagulation time (INR/aPTT), one-stage aPTT-based factor activity levels, factor X chromogenic factor activity assays, and Bethesda assay for inhibitor assessment [14]. The effect, however, could be minimized with serial dilutions [15].

The standard management of acquired hemophilia A consists of a three-prong approach with acute bleed control, with either a bypassing agent or the use of recombinant porcine FVIII (rpFVIII), immunosuppression to eliminate the autoinhibitors, and management of the underlying cause. We did not pursue the use of bypassing agents or rpFVIII as her bleeding remained stable, and we were concerned by the patient’s history of a recent pulmonary embolism and the potential for bypassing agents to increase the risk for a thromboembolic adverse event [16]. The rate of thromboembolic adverse events is similar between activated prothrombin complex concentrates (aPCCs) and recombinant activated Factor VII (rFVIIa), with 15.4 events per 1,000 aPCC-exposed patient-years vs 18.2 events per 1,000 rFVIIa-exposed patient-years, but concomitant use of both agents increases the thrombotic risk to 29.7 events per patient-year [17]. The thrombotic risk for the recombinant porcine FVIII is seemingly lower with no reported events in the multi-center phase 2/3 open-label clinical trial, which led to its commercial availability [18]. As such, we would have favored using rpFVIII for bleeding management if required after ruling out significant cross-reacting inhibitors to rpFVIII [19]. Another emerging therapeutic option for acquired hemophilia A is emicizumab. Emicizumab is a bispecific, FVIII-mimetic therapeutic antibody administered subcutaneously and has been shown to reduce the need for bypassing agents and immunosuppression in acquired hemophilia A patients [20]. Further elucidation regarding emicizumab’s efficacy, dosing, and thromboembolic risk is still needed, but it remains an important new hemostatic therapy for consideration [20].

## Conclusions

In summary, this case illustrates the need for a high index of suspicion for acquired hemophilia A and proper utilization of coagulation assays in a patient presenting with unexpected recurrent bleeding while on anticoagulation with no prior personal or family history of bleeding. The need for therapeutic LMWH led to further delays in the patient’s eventual diagnosis, contributing to significant morbidity and healthcare resource utilization. Furthermore, care must be taken when conducting and interpreting coagulation assays to avoid diagnostic errors.

## References

[1] Pai M (2021). Acquired hemophilia A. Hematol Oncol Clin North Am.

[2] Kruse-Jarres R, Kempton CL, Baudo F (2017). Acquired hemophilia A: updated review of evidence and treatment guidance. Am J Hematol.

[3] Webert KE (2012). Acquired hemophilia A. Semin Throm Hemost.

[4] Miller CH (2018). Laboratory testing for factor VIII and IX inhibitors in haemophilia: a review. Haemophilia.

[5] Beyth RJ, Landefeld CS (1995). Anticoagulants in older patients: a safety perspective. Drugs Aging.

[6] van Rein N, Biedermann JS, van der Meer FJ (2017). Major bleeding risks of different low-molecular-weight heparin agents: a cohort study in 12 934 patients treated for acute venous thrombosis. J Thromb Haemost.

[7] Witt DM, Nieuwlaat R, Clark NP (2018). American Society of Hematology 2018 guidelines for management of venous thromboembolism: optimal management of anticoagulation therapy. Blood Adv.

[8] Rodeghiero F, Pabinger I, Ragni M (2019). Fundamentals for a systematic approach to mild and moderate inherited bleeding disorders: an EHA consensus report. Hemasphere.

[9] Thomas O, Lybeck E, Strandberg K, Tynngård N, Schött U (2015). Monitoring low molecular weight heparins at therapeutic levels: dose-responses of, and correlations and differences between aPTT, anti-factor Xa and thrombin generation assays. PLoS One.

[10] Samuelson BT, Cuker A, Siegal DM, Crowther M, Garcia DA (2017). Laboratory assessment of the anticoagulant activity of direct oral anticoagulants: a systematic review. Chest.

[11] Armitage J, Ashcraft J, Kim A, Kaplan HS (1995). An approach to factor assays in patients with strong lupus anticoagulants. Clin Appl Thromb/Hemost.

[12] Chandler WL, Ferrell C, Lee J, Tun T, Kha H (2003). Comparison of three methods for measuring factor VIII levels in plasma. Am J Clin Pathol.

[13] Sahud MA, Pratt KP, Zhukov O, Qu K, Thompson AR (2007). ELISA system for detection of immune responses to FVIII: a study of 246 samples and correlation with the Bethesda assay. Haemophilia.

[14] Scheres LJ, Lijfering WM, Middeldorp S, Cheung YW, Barco S, Cannegieter SC, Coppens M (2018). Measurement of coagulation factors during rivaroxaban and apixaban treatment: results from two crossover trials. Res Pract Thromb Haemost.

[15] Douxfils J, Mullier F, Loosen C, Chatelain C, Chatelain B, Dogné JM (2012). Assessment of the impact of rivaroxaban on coagulation assays: laboratory recommendations for the monitoring of rivaroxaban and review of the literature. Thromb Res.

[16] Shapiro AD, Mitchell IS, Nasr S (2018). The future of bypassing agents for hemophilia with inhibitors in the era of novel agents. J Thromb Haemost.

[17] Jalowiec KA, Andres M, Taleghani BM (2020). Acquired hemophilia A and plasma cell neoplasms: a case report and review of the literature. J Med Case Rep.

[18] Kruse-Jarres R, St-Louis J, Greist A (2015). Efficacy and safety of OBI-1, an antihaemophilic factor VIII (recombinant), porcine sequence, in subjects with acquired haemophilia A. Haemophilia.

[19] Türkantoz H, Königs C, Knöbl P (2020). Cross-reacting inhibitors against recombinant porcine factor VIII in acquired hemophilia A: data from the GTH-AH 01/2010 study. J Thromb Haemost.

[20] Knoebl P, Thaler J, Jilma P, Quehenberger P, Gleixner K, Sperr WR (2021). Emicizumab for the treatment of acquired hemophilia A. Blood.

